# *Streptococcus salivarius* Prosthetic Joint Infection following Dental Cleaning despite Antibiotic Prophylaxis

**DOI:** 10.1155/2019/8109280

**Published:** 2019-04-21

**Authors:** Lyra B. Olson, Daniel J. Turner, Gary M. Cox, Christopher J. Hostler

**Affiliations:** ^1^Duke University School of Medicine, Durham, USA; ^2^Department of Medicine, Duke University School of Medicine, Durham, USA; ^3^Department of Medicine, Division of Infectious Diseases, Duke University School of Medicine, Durham, North Carolina, USA; ^4^Infectious Disease Section, Durham VA Health Care System, Durham, USA

## Abstract

We present the case of a 92-year-old man with septic arthritis of a prosthetic hip joint due to *Streptococcus salivarius* one week following a high-risk dental procedure despite preprocedure amoxicillin. *S. salivarius* is a commensal bacterium of the human oral mucosa that is an uncommon cause of bacteremia. *S. salivarius* has previously been described as a causative agent of infective endocarditis and spontaneous bacterial peritonitis but was only recently recognized as a cause of prosthetic joint infection. This case highlights the potential pathogenicity of a common commensal bacteria and the questionable utility of prophylactic antibiotics before dental procedures to prevent periprosthetic joint infections.

## 1. Introduction

As life expectancy increases and the baby boomers reach retirement, joint replacements have become an increasingly common procedure to improve the quality of life of individuals living with osteoarthritis. An estimated 2.5 million Americans are living with artificial hip joints, and over 200,000 join their ranks annually [1].

Periprosthetic joint infection (PJI) is a rare but serious complication of joint replacement. Estimated rates of PJI following total hip replacement are 0.5–1.6% [2]. These infections are classified into early (0–3 months postoperatively), delayed (3–12 months postoperatively), or late (greater than 12 months postoperatively). Intraoperative contamination is the most common cause of early and delayed PJI, while hematogenous seeding is most often responsible for late infection. The majority of late-onset PJI are caused by *Staphylococcus aureus* and *Staphylococcus epidermis* (57%), though about 2% of late PJI are attributable to viridans group streptococci (VGS), a diverse class of alpha-hemolytic commensal bacteria found in the human GI and GU tracts [3]. VGS are divided into 5 major groups: *S. anginosus* group, *S. mitis* group, *S. mutans* group, *S. salivarius* group, and *S. sanguinis* group [4]. Though *S. salivarius* has been implicated in spontaneous bacterial peritonitis [5] and infective endocarditis [6, 7], it has only recently been reported as the pathogenic agent in prosthetic joint infection [8]. The case presented below identifies *S. salivarius* as an uncommon commensal cause of late prosthetic joint infection, discusses management of late prosthetic joint infection due to *Streptococcus*, and serves to remind clinicians that prophylactic antibiotics before dental procedures is neither recommended nor efficacious in the prevention of periprosthetic joint infection in the vast majority of immunocompetent patients.

## 2. Case Presentation

A 92-year-old man with osteoarthritis and a remote history of right total hip replacement and bilateral total knee replacements presented with a two-day history of severe right hip pain accompanied by nausea, chills, and fatigue. He was unable to bear weight due to pain, and range of motion in his right hip was severely limited. He denied recent trauma or pain in other joints but had undergone routine dental cleaning one week prior to presentation. He had received 2 gm of amoxicillin immediately prior to his dental procedure. He presented afebrile, mildly hypotensive, and was noted to have exquisite tenderness with passive movement of the right leg and reduced range of motion secondary to pain. He had full range of motion and strength distally. He had a leukocytosis of 15,600 WBC per milliliter. Aspiration of right hip joint revealed cloudy fluid with WBC 68,000 cells per milliliter. The patient was resuscitated with IV fluids and started on vancomycin and piperacillin-tazobactam. On the third hospital day, he underwent prosthetic joint drainage and washout with retention of liners. Joint aspirate culture grew Gram-positive cocci in chains, which we subsequently identified by matrix-assisted desorption ionization time-of-flight mass spectrometry (MALDI-TOF MS) as *S. salivarius*. We narrowed antibiotic therapy to ceftriaxone to complete six total weeks of intravenous therapy, after which he was treated with amoxicillin for additional six weeks. At this point, the patient discontinued his oral antibiotics rather than complete a full three months of oral therapy or remain on long-term suppression.

Five months after completion of the above therapy, the patient presented to the ED with hip and back pain following a mechanical fall at home. He was found to have a fracture of L4 and was admitted. On hospital day 5, he was found to have an occult superior dislocation of the R femoral head on CT and an ESR of 71 and CRP of 12.3. He was taken to the OR for single-stage full revision of his prosthesis the following day. One of the three intraoperative cultures had light growth of coagulase-negative staph and *S. salivarius*, though no WBCs were seen and gram stain was negative. He was treated with 6 weeks of vancomycin and remains on lifelong antibiotic suppression.

## 3. Discussion

Here, we present a case of a late PJI of the hip caused by *S. salivarius,* a rarely reported cause of infection originating from the oral mucosa, following a dental cleaning. There are several potential reasons that *S. salivarius* has only recently been recognized in the literature as a cause of PJI. Compared to other species in the VGS family, which already make up less than 2% of PJI, *S. salivarius* is the least virulent and is often regarded as a contaminant in culture. In one prospective study, less than a third of laboratory-confirmed *S. salivarius* bacteremia were considered clinically significant, compared with 97% of bacteremia with the closely related *S. bovis* [6]. In the largest retrospective study of streptococcal PJIs to date and one of the only other studies to identify *S. salivarius* as a cause of PJI, only 4 cases of nearly 500 streptococcal periprosthetic joint infections were attributed to *S. salivarius* [8].

On a more technical basis, many hospital laboratories historically have not speciated VGS organisms, as speciation through classic biochemical testing is time consuming and of only moderate reliability [9]. In the past 5 years, however, widespread use of MALDI-TOF MS has enhanced the ability of clinical microbiology labs to accurately identify infectious agents [10], including speciation of VGS [11, 12]. This technology enables rapid, consistent identification of microorganisms through comparison of spectral data of cellular proteins from whole-cell patient samples with FDA-approved database of reference spectral signatures of hundreds of species of bacteria and fungi. As VGS speciation through MALDI-TOF MS becomes a mainstream component of clinical microbiology, *S. salivarius* will likely be recognized as an infrequent but not unanticipated cause of PJI.

Consistent speciation of VGS will improve the care of patients with PJIs through tailored antibiotic regimens and improved study of strain-specific risk factors and outcomes. This is especially important as PJI caused by VGS may be increasing as a proportion of PJIs overall [13]. Identification of species-specific infection trends will enable more detailed retrospective study to identify if the uptick in VGS PJI is driven by the group as a whole or a subset of the family. In addition, several studies have indicated that VGS species differ in antibiotic susceptibility patterns, with *S. salivarius* having the lowest rates of antibiotic resistance and *S. mitis* demonstrating the most beta-lactam resistance [3, 14].

In the case at hand, management of this patient's PJI with debridement, antibiotics, and implant retention (DAIR) was a reasonable choice, given the patient's age and medical comorbidities, the stability of his prosthetic joint, and the short duration of infectious symptoms prior to presentation. Per the 2012 IDSA guidelines, patients should be considered for DAIR if the joint is less than 30 days old or if the duration of infectious symptoms is less than 21 days in the absence of a draining sinus tract [15]. Extended treatment with beta-lactam antibiotics, like this patient received, is independently associated with improved outcomes for streptococcal PJI treated by DAIR [8].

Acknowledging that the patient's clinical presentation at time of recurrence is attributable to his mechanical fall, his elevated ESR and CRP and isolation of *S. salivarius* from one operative sample five months after completion of initial therapy suggest a possible indolent recurrence of PJI. A large retrospective cohort study of streptococcal PJI managed by DAIR has a failure of treatment in 42.1% of cases, where failure was defined by relapse or persistence of infection, need for salvage therapy, or death related to infection [8]. Even in cases where IDSA criteria were met for washout, the failure rate was still 37%. This patient had several risk factors for recurrence of his PJI after DAIR management. Initial presentation with late PJI is an independent predictor for failure of DAIR after completion of therapy, as is retention of removable components [8], which was unavoidable given the age of this patient's prosthesis.

In the effort to prevent this difficult complication of joint replacement, many patients, including the patient in this case, are directed to take prophylactic antibiotics prior to dental procedures. Indeed, dental procedures are associated with transient bacteremia, including VGS bacteremia [16], and the majority of late PJI arises from hematologic bacterial seeding of the prosthesis [17]. Preprocedure antibiotics reduce the frequency and duration of bacteremia after dental procedures by up to 50% [16, 18]. However, many studies have failed to demonstrate any increased risk of PJI as a result of dental procedure, with or without antibiotic prophylaxis [19–23]. Furthermore, transient bacteremia also occurs with activities of daily living like brushing teeth, flossing, and even chewing [24–26], although the frequency and degree of bacteremia associated with these activities is far less than with dental cleaning or extractions [16]. In the case we present, it remains possible that the patient became bacteremic from tooth brushing or chewing, though it is more likely a result of his recent dental cleaning. The most recent clinical practice guideline from the AAOS reflect the current data and recommend against routine use of prophylactic antibiotics for dental procedures in patients with joint replacement [27]. Nevertheless, it remains common practice for orthopedists to recommend prophylactic antibiotics indefinitely following joint replacement [28, 29].

If patients and physicians seek to reduce the risk of hematogenous PJI from oral flora, attention could be turned to improving oral hygiene. The frequency and magnitude of bacteremia following dental procedures is increased in patients with periodontal disease as compared to those with healthy gums [24]. While there is no evidence directly linking better oral health with reduced risk of PJI, dental hygiene is a low-cost intervention that improves both oral health and individual quality of life. At this time, the AAOS has a consensus recommendation for the improvement or maintenance of appropriate oral hygiene as a means of potentially reducing bacteremia [27].

In conclusion, late PJI remains an uncommon but challenging complication of joint replacement. Commensal organisms like *S. salivarius* rarely cause infections even in immunocompetent individuals. Improved identification of causative organisms with microbiologic lab techniques like MALDI-TOF MS will enable us to improve algorithms of care for different infectious agents. Clinicians continue to prescribe antibiotic prophylaxis prior to dental procedures despite the lack of evidence supporting this practice. Increased awareness of current guidelines and data would save health care dollars and prevent unnecessary antibiotic exposure for millions of patients.
